# Colorectal cancer risk stratification using a polygenic risk score in symptomatic primary care patients—a UK Biobank retrospective cohort study

**DOI:** 10.1038/s41431-024-01654-3

**Published:** 2024-08-01

**Authors:** Bethan Mallabar-Rimmer, Samuel W. D. Merriel, Amy P. Webster, Leigh Jackson, Andrew R. Wood, Matthew Barclay, Jessica Tyrrell, Katherine S. Ruth, Christina Thirlwell, Richard Oram, Michael N. Weedon, Sarah E. R. Bailey, Harry D. Green

**Affiliations:** 1 Department of Clinical and Biomedical Sciences, University of Exeter, Exeter, UK; 2 Division of Population Health, Health Services Research & Primary Care, University of Manchester, Manchester, UK; 3 Department of Behavioural Science & Health, Institute of Epidemiology & Health Care, University College London, London, UK; 4 Bristol Medical School, University of Bristol, Bristol, UK; 5 Department of Health and Community Sciences, University of Exeter, Exeter, UK

**Keywords:** Genetic testing, Cancer screening, Risk factors, Colorectal cancer, Cancer genomics

## Abstract

Colorectal cancer (CRC) is a leading cause of cancer mortality worldwide. Accurate cancer risk assessment approaches could increase rates of early CRC diagnosis, improve health outcomes for patients and reduce pressure on diagnostic services. The faecal immunochemical test (FIT) for blood in stool is widely used in primary care to identify symptomatic patients with likely CRC. However, there is a 6–16% noncompliance rate with FIT in clinic and ~90% of patients over the symptomatic 10 µg/g test threshold do not have CRC. A polygenic risk score (PRS) quantifies an individual’s genetic risk of a condition based on many common variants. Existing PRS for CRC have so far been used to stratify asymptomatic populations. We conducted a retrospective cohort study of 50,387 UK Biobank participants with a CRC symptom in their primary care record at age 40+. A PRS based on 201 variants, 5 genetic principal components and 22 other risk factors and markers for CRC were assessed for association with CRC diagnosis within 2 years of first symptom presentation using logistic regression. Associated variables were included in an integrated risk model and trained in 80% of the cohort to predict CRC diagnosis within 2 years. An integrated risk model combining PRS, age, sex, and patient-reported symptoms was predictive of CRC development in a testing cohort (receiver operating characteristic area under the curve, ROCAUC: 0.76, 95% confidence interval: 0.71–0.81). This model has the potential to improve early diagnosis of CRC, particularly in cases of patient noncompliance with FIT.

## Introduction

Colorectal cancer (CRC) is the second most common cause of cancer mortality in the UK and worldwide [1, 2]. In the UK, 37–41% of CRC cases are diagnosed at an early stage (Dukes stage A) [3]. Diagnosis at an early stage improves prognosis, and is a research priority for CRC [4, 5] and an NHS target for all cancers [6]. Since 67% of UK CRC cases are diagnosed following a primary care presentation [7], expedited diagnosis in this setting has potential to improve patient outcomes.

### Current diagnostic practice

Patients referred urgently for suspected CRC often receive a computed tomography scan or endoscopy procedure (e.g., colonoscopy, flexible sigmoidoscopy) [8]; around 10% of patients referred urgently for suspected CRC received a CRC diagnosis [9]. In 2021, 540,867 colonoscopies were performed in the UK, of which 90.88% were for diagnostic or therapeutic purposes (the remaining 9.12% were for screening) [10]. Colonoscopies have high cost [11], detrimental environmental impact [12], and may cause patients avoidable discomfort and distress [13]. There is a clear need for improved targeting of CRC diagnostic procedures to patients most at risk. Improving CRC risk assessment could improve early diagnosis rates and reduce the volume of colonoscopies, thereby reducing burden on patients, clinicians and healthcare providers [14].

National Institute for Health and Care Excellence (NICE) guidelines, published in August 2023, recommend triaging patients with bowel symptoms for CRC diagnosis using the quantitative faecal immunochemical test (FIT) for blood in stool [15]. NICE estimate this will prevent 94,291 colonoscopies per year [16]. FIT is offered based on symptoms which vary by age (abdominal mass, change in bowel habit, or iron-deficiency anaemia for patients of all ages; abdominal pain and unexplained weight loss if age 40+; rectal bleeding and one of abdominal pain or weight loss if 50+; anaemia if 60+) [15]. Extensive evidence supports FIT as highly sensitive and specific for CRC risk [17–19]. However, FIT uptake varies by age, sex, ethnicity and socioeconomic status, with a 6.4–16.2% noncompliance rate in-clinic [15, 20]. An evaluation of FIT in clinic showed that 43 (6.97%) of 618 patients with a FIT result over the symptomatic 10 ug/g threshold had CRC [21]. This study presents a novel method of patient risk assessment in primary care using genetics, symptoms, and patient characteristics, with potential to complement existing methods such as FIT in cases of noncompliance or uncertain results.

### Genetic and integrated risk models to stratify patient risk of CRC

Risk models including both genetic and environmental risk factors have more power to discriminate between CRC cases and controls, compared to models only including environmental risk factors [22].

A polygenic risk score (PRS) quantifies an individual’s genetic risk of a condition based on many variants [23]. To date, PRS for CRC have screened for CRC risk in asymptomatic populations [24–27]. CRC symptoms can be non-specific for cancer—e.g., abdominal pain, weight loss—making it challenging for identify patients who would benefit from a referral [28]. It has previously been shown that a PRS can stratify symptomatic patients according to risk of developing prostate cancer within a 2-year window [29]. Therefore, in this study, an integrated risk model (IRM) was developed which combines information about patient symptoms with a PRS and other risk factors, to predict which of a cohort of symptomatic patients are most at risk of CRC in the next 2 years.

### Aim

The aim of this study was to build an IRM—including environmental risk factors, patient demographics, symptoms, and a PRS—to predict which patients with CRC symptoms will be diagnosed with CRC within 2 years of their first presentation to primary care with a symptom. The intended use of the IRM is to support clinicians in identifying symptomatic patients suitable for referral for suspected CRC.

### Reporting standards

This report has been written in line with the Polygenic Risk Score Reporting Standards published by the Polygenic Score Catalogue and the Clinical Genome Resource Complex Disease Working Group [30].

## Subjects and methods

### Study design and cohort

This was a retrospective cohort study using primary data from UK Biobank (UKBB). UKBB is a database of 500,000 individuals recruited at ages 40–69, between 2006 and 2010, described extensively elsewhere [31]. General practice (GP) records between 1938 and 31 August 2017 were available for ~230,000 participants [32, 33].

All analysis in this study was completed on the UKBB Research Analysis Platform on DNAnexus, using R coding language version 4.1.1. The study cohort included UKBB participants with at least one CRC symptom in their GP record at or after the age of 40. Symptomatic participants were identified by searching UKBB GP records for Read v2 and v3 codes [34] for the following CRC symptoms: abdominal mass, abdominal pain, appetite loss, change in bowel habit, iron deficiency, low haemoglobin, rectal bleeding, weight loss. Low haemoglobin was defined as <11 grams per decilitre (g/dl) in females and <13 g/dl in males (using self-reported sex of patients) [35]. The number of Read codes for each CRC symptom, and the number of participants in the final study cohort with each symptom, is listed in Supplementary Table 1. For the full list of Read codes, see the ‘Data Availability’ section of this manuscript.

**Fig. 1:**
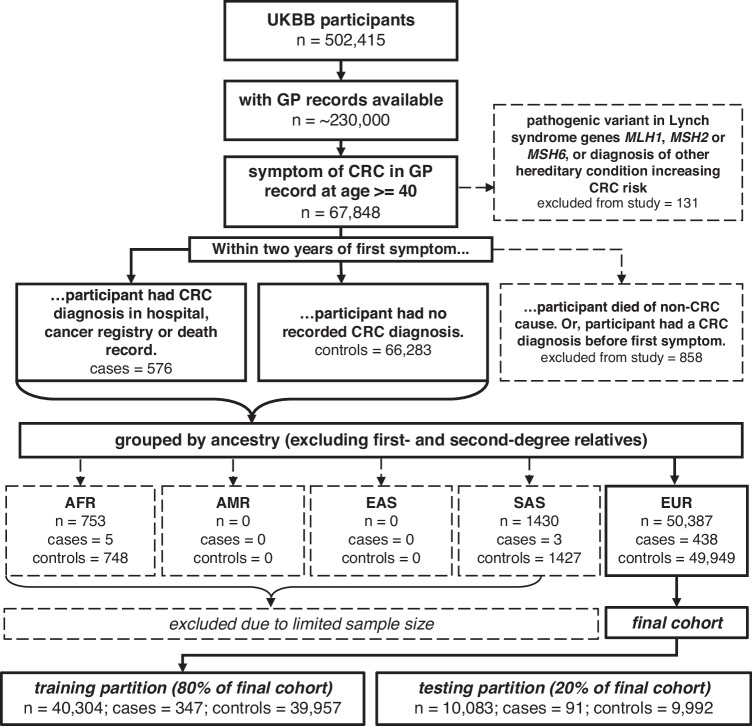
Flowchart of study design. Participants with a CRC symptom in their GP record at age 40+ were included in the study. Cases had a CRC diagnosis within 2 years of first symptom, whereas controls did not. Excluded participants: died within 2 years of first symptom (not from CRC), had CRC before first symptom, had non-European ancestry (excluded due to limited case numbers), were related to the first- or second-degree, had a pathogenic variant in Lynch syndrome genes *MLH1*, *MSH2* or *MSH6*, or were diagnosed in primary care records with one of: familial adenomatous polyposis, Gardner syndrome, Turcot syndrome, hereditary flat adenoma syndrome, hereditary nonpolyposis CRC, hereditary mixed polyposis syndrome, or the hamartomatous polyposis syndromes. Only primary care records were used to find hereditary CRC syndrome diagnoses, as the ICD-10 codes these conditions fall under are not specific to the conditions, also encompassing any benign neoplasm of the digestive system. AFR African, AMR Admixed America, CRC colorectal cancer, EAS East Asian, EUR European, GP general practice, SAS South Asian, UKBB UK Biobank.

‘Index date’ refers to the date of a participant’s first recorded CRC symptom, at or after age 40. The age threshold of 40 when searching participants’ symptom records was used because very few UKBB participants were diagnosed with CRC younger than this (Supplementary Fig. 1). The cohort was divided into cases, who had a CRC diagnosis within 2 years of index date, or controls, with no CRC in that period. To ensure our cohort matched the real life population facing primary care, we did not match controls by any clinical features. A CRC diagnosis was defined by searching for ICD-10 codes in participants’ Cancer Registry, Hospital Inpatient Data, and Death records, or for Read codes describing CRC in GP records (see ‘Data Availability’). The ICD-10 codes used referred to malignancies of the colon (C18), rectosigmoid junction (C19), and rectum (C20). CRC was defined as right-sided if located between the caecum and hepatic flexure, or left-sided between the splenic flexure and rectum. Participants were excluded from the study if they had CRC before the index date, or if they died of a cause other than CRC within 2 years, as it cannot be determined whether they would have developed CRC. Individuals with pathogenic variants associated with increased risk of CRC in Lynch syndrome genes *MLH1, MSH2* and *MSH6* [36] or a diagnosed hereditary CRC syndrome, were excluded. Study design is summarised with a flowchart in Fig. 1.

### Ancestry

UKBB participants with European (*n* = 379,768), South Asian (*n* = 9455), African (*n* = 7440), East Asian (*n* = 2481), and Admixed American (*n* = 467) ancestry, excluding individuals related to the first or second degree, were determined using principal components analysis and KING kinship [37]. Following filtering for availability of GP records and presence of a CRC symptom, the European cohort included 438 cases and 49,949 controls, the African cohort 5 cases and 748 controls, the South Asian cohort 3 cases and 1427 controls, and there were no cases or controls with either East Asian or Admixed American ancestry. Due to this limited sample size, the PRS and IRM could only be developed and assessed for predictivity in the European cohort. However, PRS distribution in UKBB was assessed across all five ancestral superpopulations (see Results).

### Genetic data

UKBB participants were genotyped at ~850,000 variants with the UKBB Axiom Array, and a further ~96 million variants were imputed (steps detailed by Bycroft et al.) [31] PRS construction in this study used participant genotypes imputed by the Wellcome Trust Centre for Human Genetics, version 3 in UKBB [38].

### PRS construction

A PRS quantifying participants’ genetic risk of CRC was calculated using 205 genetic variants associated with long-term risk of developing CRC, and their betas (the log odds ratio of the association between variant and phenotype), published by Fernandez-Rozadilla et al. in recent genome-wide meta-analysis [39]. The meta-analysis included 100,204 cases with a diagnosis of CRC, and 154,587 controls without, from ~90 studies. Inclusion criteria varied by study. 73% of individuals in the meta-analysis were European and 27% were East Asian, mostly matching the European ancestry of our cohort. 95.2% of cases and 86.9% of controls were not from UKBB, reasonably avoiding overfitting with UKBB data.

Four variants, described in Supplementary Fig. 2, were excluded from PRS construction. For each participant in UKBB, a PRS was calculated by scoring dosage (the expected/predicted genotype following imputation) at each of the remaining 201 genetic variants, multiplying each score by the beta for the variant, and summing these values.

### Model validation

The cohort was partitioned into a training dataset for model building (containing a random 80% of the cohort with age, sex and case ratio preserved) and a testing dataset comprising the remaining 20% of participants.

Testing and training datasets were further stratified into sub cohorts according to age at first symptom (40–49, 50–59, 60–69, or 70–79) and sex. Overall, the IRM including PRS was developed and evaluated in a total of 14 patient groups (4 grouped by age, 2 by sex, 8 by both age and sex) as well as the full cohort. Age, sex and case/control distributions are summarised in Table 1 for the non-partitioned full cohort (Supplementary Table 2 for sub cohorts and training/testing partitions).

**Table 1:** Age, sex, and numerical variable distribution of cases and controls in the full cohort.

	Cases	Controls	Full Cohort
*N* (% of cohort)	438 (0.87%)	49,949 (99.13%)	50,387 (100%)
Sex (patient self-reported)			
Female	191 (43.61%)	29,830 (59.72%)	30,021 (59.58%)
Male	247 (56.39%)	20,119 (40.28%)	20,366 (40.42%)
Age at UKBB baseline/recruitment (years)			
Mean (±SD)	60.42 (±6.66)	57.75 (±7.56)	57.78 (±7.56)
Age at first CRC symptom (years)			
Mean (±SD)	59.67 (±7.44)	54.82 (±8.65)	54.86 (±8.65)
Body mass index (BMI) (kg/m^2^)			
Mean (±SD)	27.55 (±4.57)	27.73 (±4.96)	27.73 (±4.96)
Waist circumference (cm)			
Mean (±SD)	92.44 (±13.73)	90.56 (±13.71)	90.58 (±13.71)
Townsend deprivation index (TDI)			
Mean (±SD)	−1.74 (±2.9)	−1.48 (±2.94)	−1.48 (±2.94)

Distributions of these variables are reported for the full cohort and all subcohorts in Supplementary Table 2. Categorical variable distribution across all cohorts is reported in Supplementary Table 5.

*N* number of participants, *SD* standard deviation.

### Non-genetic variables and logistic regression analysis

In the training data, a total of 28 variables (PRS and 27 others derived from UKBB data-fields) were tested individually for association with CRC diagnosis within the 2-year period, using logistic regression. Variables included participant characteristics (age at first CRC symptom and sex), lifestyle variables (Townsend deprivation index, smoking, alcohol intake, and processed meat intake), health measures (body mass index, waist circumference, and whether the participant self-reported having diabetes), PRS, which symptom/s the participant reported at index date only (symptoms from follow-up GP visits were not included), and family history (i.e., whether the participant self-reported a history of bowel cancer in either parent). The first five genetic principal components in UKBB were also included as variables, to test for confounding.

All variables were measured at baseline (UKBB recruitment), except genetic variables (genotyping data was released in 2017, imputed genotyping data in 2022) and symptoms at index/age at first symptom. Median time difference between index date and recruitment was 1603 days (±interquartile range 1956)—Supplementary Fig. 3. Variables are described in-detail in Supplementary Table 3.

All variables had values for >99% of participants (Supplementary Table 4)—any missing values were omitted from logistic regression analysis. Variables significantly associated with either the case or control group (*p* value < 1.8e−03, Bonferroni corrected threshold) were further tested for ability to predict CRC diagnosis when included in an IRM.

### IRM description and fitting

The IRM estimates the cross-sectional risk of a patient with CRC symptoms either being diagnosed with the cancer within 2 years of first symptom or not. An IRM was developed in each training dataset, for optimal performance according to two measures; receiver operating characteristic area under the curve (ROCAUC) and Akaike information criterion (AIC).

The IRM was constructed by starting with the variable with highest ROCAUC and iteratively adding variables in order of which cause the largest increase in ROCAUC. At every iteration, ROCAUC was calculated using five-fold cross validation, to avoid overfitting the IRM to the training dataset.

A second method of IRM construction involved building all possible risk models and ranking these according to AIC score—where a lower AIC score indicates a model which better fits the training dataset while excluding inessential variables. Using AIC to penalise models including more variables reduces overfitting.

IRMs constructed with the above methods were evaluated for predictivity using ROCAUC in each testing cohort.

## Results

### Participant demographics

The final cohort consisted of 50,387 participants (438 cases and 49,949 controls) who were related to no more than the third degree, had European ancestry, and had a CRC symptom recorded in their GP record between the ages of 40–79 (no UKBB participants had a CRC symptom recorded at 80 or older). Mean age at index date was 54.9. 40.5% of the cohort were male. Incidence of CRC in the cohort (percentage of cases) was 0.87% (~0.44% per year). See Table 1 for further demographics.

### Symptoms and factors associated with CRC risk

In the training partition of the full cohort, six variables were associated (*p* < 1.8e−03, Bonferroni-corrected) with increased risk of CRC diagnosis within 2 years of first symptom presentation, relative to other symptomatic patients in the cohort. These included: older age at index date; higher PRS; self-reported sex being male; smoking previously (as opposed to never having smoked or being a current smoker); rectal bleeding; and change in bowel habit.

The finding that previous smokers had increased risk of CRC within the 2-year period compared to those who reported being current smokers may indicate underlying bias or confounding in this variable; e.g. previous smokers may have smoked more heavily or for more years on average. This variable was therefore excluded from the IRM.

One variable, abdominal pain, was more prevalent in controls than cases, and was therefore associated (*p* = 4.3e−38) with decreased risk of CRC diagnosis within 2 years relative to participants reporting other symptoms. CRC incidence in participants with abdominal pain was 0.38% (~0.19% per year) in the training cohort—Supplementary Table 5—similar to annual CRC incidence in the UK of 0.13% [40]. Table 2 shows the *p* values, odds ratios, individual ROCAUC scores, and 95% confidence intervals of the seven variables associated with either the case or control group following logistic regression. Supplementary Table 6 shows the results of logistic regression for all 28 variables across all cohorts.

**Table 2:** *p* Values, odds ratios, ROCAUC, and 95% confidence intervals for all variables shown via logistic regression to be associated with the case or control group, in the training partition of the full cohort.

Risk factor/marker	*p* value	Odds ratio	ROCAUC
Rectal bleeding	2.00 × 10^−38^	4.06 (3.29–5.02) if symptom recorded at index date	0.65 (0.62–0.67)
Abdominal pain	4.30 × 10^−38^	0.22 (0.17–0.27) if symptom recorded at index date	0.68 (0.66–0.71)
Age at first CRC symptom	3.90 × 10^−24^	1.07 (1.05–1.08) per year increase	0.66 (0.64–0.69)
PRS	8.3 × 10^−13^	1.33 (1.23–1.44) per quintile increase	0.62 (0.58–0.64)
Change in bowel habit	1.50 × 10^−9^	2.32 (1.77–3.06) if symptom recorded at index date	0.55 (0.53–0.57)
Sex	1.70 × 10^−9^	1.92 (1.56–2.38) if male	0.58 (0.55–0.61)
Smoking (current, previous, or never)			
Never -> previous	0.0013	1.44 (1.15–1.8) if previous smoker	0.55 (0.52–0.57)
Never -> current	0.79	1.05 (0.72–1.53) if current smoker	0.5 (0.48–0.53)
Previous -> current	0.1	0.73 (0.5–1.07) if current smoker	0.52 (0.5–0.55)

For logistic regression results in subcohorts and both training/testing partitions, see Supplementary Table 6. 95% confidence intervals are shown in brackets. Risk factors and markers in the table are ordered according to strength of *p* value. A *p* value threshold of <1.8e−03 follows Bonferroni correction of *α* = 0.05 for 28 tests. Abdominal pain has an odds ratio <1, showing that participants with abdominal pain had decreased risk of CRC within 2 years of first symptom relative to the rest of the cohort. Additionally, results suggest that current smokers had decreased risk of CRC diagnosis within 2 years of first symptom relative to participants in the cohort who self-identified as previous smokers. This may be due to confounding; for example, it is possible that previous smokers may have smoked more heavily or for longer on average than participants who reported currently smoking.

*CRC* colorectal cancer, *PRS* polygenic risk score, *ROCAUC* receiver operating characteristic area under the curve.

### IRM construction

**Fig. 2:**
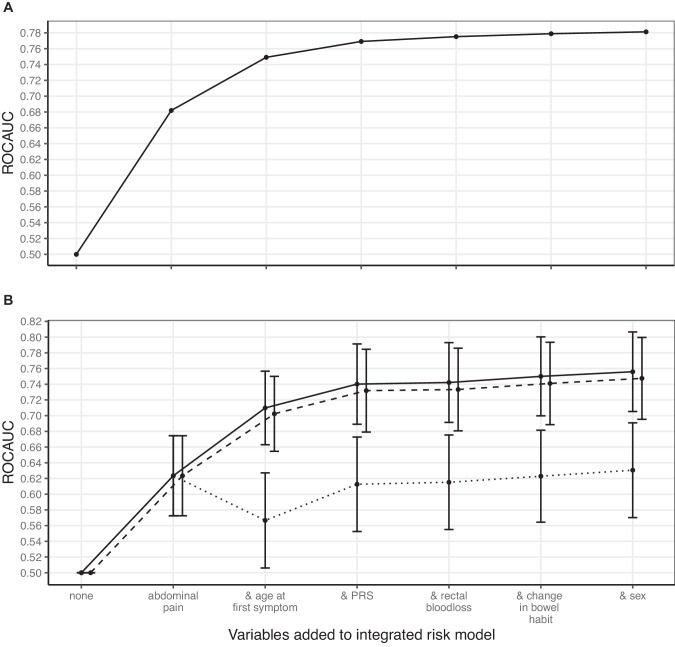
Adding variables to the IRM in order of which cause the largest increase in ROCAUC in the training dataset, and replication in the testing dataset. **A** As variables are added to the IRM, ROCAUC tends towards 0.78 in the training partition of the full cohort. Variables were added in order of which caused the greatest increase in ROCAUC. **B** Replicating the results of (**A**) in the testing partition of the full cohort. The same six variables had combined ROCAUC of 0.76 (CI95: 0.71–0.81) when predicting all cases of CRC (solid line), 0.75 (CI95: 0.70–0.80) when predicting left-sided CRC (dashed line), and 0.63 (CI95: 0.57–0.69) when predicting right-sided CRC (dotted line). However, using only age at first symptom, sex, and PRS to predict right-sided CRC had ROCAUC of 0.71 (CI95: 0.65–0.76). PRS polygenic risk score, ROCAUC receiver operating characteristic area under the curve.

The seven aforementioned variables (Table 2), minus smoking status, were used to construct an IRM in the training dataset. After applying AIC scoring to all 63 possible combinations of six variables, an IRM combining all six variables—age at first symptom, sex, PRS, abdominal pain, rectal bleeding and/or change in bowel habit—had the lowest AIC score (Table 3). The other method of IRM construction used in this study involved adding variables to the IRM in order of which caused the largest increase in ROCAUC. Figure 2 shows that, in the training dataset, all six variables added predictivity to the IRM, concurring with the results of AIC scoring.

**Table 3:** The five IRMs with lowest AIC scores in the full cohort.

IRM (number of variables)	AIC score in training dataset	ROCAUC (CI95) in training dataset	ROCAUC (CI95) in testing dataset
Age, abdominal pain, PRS, sex, rectal bleeding, change in bowel habit (6)	3616.81	0.78 (0.76–0.81)	0.76 (0.71–0.81)
Age, PRS, sex, rectal bleeding, change in bowel habit (5)	3617.15	0.78 (0.76–0.81)	0.76 (0.71–0.81)
Age, abdominal pain, PRS, rectal bleeding, change in bowel habit (5)	3630.21	0.78 (0.76–0.80)	0.75 (0.70–0.80)
Age, PRS, rectal bleeding, change in bowel habit (4)	3630.84	0.78 (0.76–0.80)	0.75 (0.70–0.80)
Age, abdominal pain, PRS, sex, rectal bleeding (5)	3632.96	0.78 (0.76–0.80)	0.75 (0.70–0.80)

63 IRMs were constructed using all possible combinations of the following six variables: age at first symptom, sex, PRS, and whether the patient reported symptoms of abdominal pain, change in bowel habit, and/or rectal bleeding at index date. AIC scoring was applied to all 63 models. Models which better fit the dataset, while excluding extraneous variables, have lower AIC scores. An individual AIC score cannot be meaningfully interpreted without comparison to other AIC scores. AIC scores across all 63 models ranged from 3616.81 to 4896.90 (range = 1280.09). The five IRMs with lowest AIC scores in each subcohort are shown in Supplementary Table 7. Variables significantly associated (logistic regression *p* < 1.8e−03, Bonferroni-corrected threshold) with either the case or control group vary by sub cohort. Only variables with significant association were used to construct IRMs. Therefore, the IRMs tested using AIC scoring are different in each sub cohort.

*AIC* Akaike information criterion, *CI95* 95% confidence interval, *IRM* integrated risk model, *PRS* polygenic risk score, *ROCAUC* receiver operating characteristic area under the curve.

### IRM validation

Predictivity of the six-variable IRM was evaluated in the testing partitions of the full cohort and sub cohorts. The IRM had a ROCAUC in the full testing cohort of 0.76, with a 95% confidence interval (CI95) of 0.71–0.81. Mean probability of each participant being a case was 0.85% (standard deviation (SD): 1.11%, range: 0.03–17.81%). Youden’s *J* statistic was optimised with a risk threshold of 0.59% (sensitivity 83.5%, specificity 60.7%). In Table 4 we report diagnostic accuracy statistics (sensitivity, specificity, PPV, NPV, and Youden’s *J* statistic) for predicted risk thresholds 0.59%, 1%, 2%, and 3%.

**Table 4:** Diagnostic statistics on the testing cohort.

Threshold	Specificity	Sensitivity	PPV	NPV	Youden’s
0.59%	0.607	0.835	0.019	0.998	0.442
1%	0.752	0.593	0.021	0.995	0.345
2%	0.894	0.352	0.029	0.993	0.246
3%	0.949	0.209	0.036	0.992	0.158

Diagnostic statistics estimated for risk thresholds of 3, 2, 1, and 0.59%. 3% risk is the threshold used by NICE to guide referrals, although 2 or 1% are preferred by patients, and 0.59% optimises Youden’s *J* statistic. All diagnostic statistics calculated on the testing cohort.

ROCAUC was higher when predicting left-sided (0.75, CI95 = 0.70–0.80) than right-sided CRC (0.63, 0.57–0.69). ROCAUC was equivalent in both (0.71, CI95 = 0.65–0.76) if excluding symptom variables (abdominal pain, rectal bleeding, and change in bowel habit) from the IRM, reflecting the fact right-sided CRC presents with different symptoms.

Mean ROCAUC across testing sub cohorts divided by age and sex was 0.75 (SD: 0.06). The IRM had highest ROCAUC in participants aged 50–59 (mean: 0.76, SD: 0.04) and 70–79 (mean: 0.79, SD: 0.02), although confidence intervals were wide in the 40–49 and 70–79 subcohorts due to small sample sizes (*N* = 3145 and 475 respectively)—Supplementary Fig. 4. When splitting the full cohort by sex but not age, ROCAUC was slightly higher in male (0.75, CI95 = 0.69–0.82) than female (0.74, CI95 = 0.66–0.83) participants. Supplementary Table 7 shows results of IRM evaluation across cohorts.

### PRS evaluation

The PRS, based on variants derived in [39], was evaluated in 100% of each (sub)cohort, rather than a 20% testing partition. The PRS had a ROCAUC of 0.62 (CI95 = 0.59–0.69) in the full cohort, showing moderate ability to discriminate between symptomatic participants with or without CRC. ROCAUC was equivalent when predicting left-sided CRC, right-sided CRC, and both.

Supplementary Fig. 5 shows PRS distribution is higher in cases than controls in all cohorts (including training and testing partitions) except female participants aged 40–49. However, logistic regression results (Supplementary Table 6) showed no evidence of an effect in participants aged 40–49 or 70–79. Conversely, Supplementary Fig. 6 shows that PRS increases predictivity of the IRM in most cohorts; particularly in male participants aged 60–79, the PRS alone is more predictive than the IRM without PRS.

**Fig. 3:**
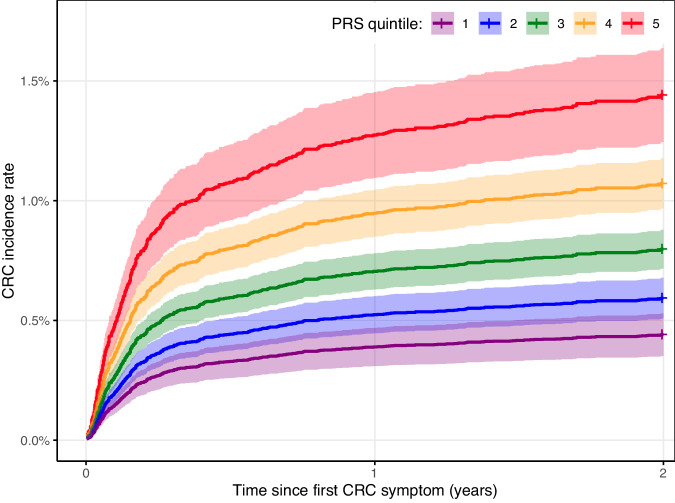
Cumulative hazard plot showing participants’ risk of CRC over 2 years from date of first symptom, stratified by PRS quintile. In the full, non-partitioned cohort, 1.45% (CI95:1.25–1.63%) of participants in the highest PRS risk quintile were diagnosed with CRC after 2 years, vs. 0.42% (CI95:0.35–0.53%) of participants in the lowest quintile. Quintile cut-offs were calculated in the entire cohort, of which 99.13% were controls. CI95 95% confidence interval, CRC colorectal cancer, PRS polygenic risk score.

Figure 3 shows that in the full cohort, 1.45% of participants with a PRS in the highest quintile were diagnosed with CRC within 2 years of first reported symptom, compared to 0.42% of participants with a PRS in the lowest quintile.

Predictivity of the PRS could not be assessed in non-European UKBB participants due to insufficient sample size. However, mean PRS distribution differed between ancestries in 399,454 African, Admixed American, East Asian, European, and South Asian UKBB participants (Supplementary Fig. 7), being higher in Europeans. This excludes participants who were related or did not cluster into an ancestral population as described in [37], or for whom a PRS could not be calculated.

## Discussion

In this study, we developed and tested a PRS and IRM in a symptomatic primary care population, for the prediction of CRC within 2 years of a patient first reporting a symptom. Results showed that a PRS including 201 genetic variants can predict which symptomatic patients have either left- or right-sided CRC with moderate accuracy (ROCAUC = 0.62, CI95 = 0.59–0.65). To our knowledge, this is the first study to use a PRS to predict short-term CRC risk in symptomatic patients. However, numerous published PRS for long-term CRC risk have similar ROCAUCs of 0.629–0.631 in European populations [25–27]. Additionally, Thomas et al. [41] demonstrated the clinical utility of a PRS across ancestries, to stratify individuals for screening according to short-term CRC risk.

In total, 1.45% of participants with PRS in the highest quintile and 0.42% in the lowest quintile were diagnosed with CRC within 2 years. In a 2014 study of patient perspectives, 81% of respondents chose to be investigated for CRC at a risk of 1%, indicating that this information is valuable to patients [42]. The overall incidence rate in our cohort is higher than annual CRC incidence in the UK (0.13% in 2017, non-age standardised) [40], likely because this study assesses a middle-aged, symptomatic population. Increased PRS was associated with higher risk of CRC diagnosis within the 2 years in all groups, except participants aged 40–49 and 70–79.

The IRM developed in this study combined measures of PRS, age at symptom presentation, sex, and the presence of symptoms associated with CRC (abdominal pain, rectal bleeding, or change in bowel habit). The IRM was predictive of CRC diagnosis within 2 years of first reported symptom, with a ROCAUC of 0.76 (CI95 = 0.71–0.81) in a cohort of European participants between the ages of 40–79. The IRM excluding the PRS has ROCAUC of 0.73 (CI95 = 0.69–0.78), supporting Kachuri et al.’s findings [22] that genetic variables increase risk model predictive power, although there is an overlap in confidence intervals (Supplementary Fig. 6).

IRM predictivity was robust across participant groups stratified by age and sex, with ROCAUC ranging from 0.65–0.84 (mean: 0.75, SD: 0.06). Predictivity compares favourably to existing IRMs for population screening (no models predicting CRC risk in a symptomatic population were available for comparison), with reported ROCAUC values between 0.57 and 0.78 (not including genetic biomarker tests, with ROCAUCs up to 0.88) [43].

### Limitations

Neither the PRS nor IRM outperform FIT, which has ROCAUC 0.95 and remains the gold standard for assessment of patient risk [44]. FIT is a disease marker, which indicates if a cancer is present or not, whereas PRS and IRM indicate the likelihood of cancer developing. Patient noncompliance with FIT is 6.4–16.2% in primary care; risk assessment tools without FIT are needed in that setting [20]. The IRM could be used to calculate a patient’s risk of CRC rapidly in the primary care setting, if enough information (age, sex, symptoms, and genotyping data) were available, enabling more rapid investigation of high-risk individuals. The clinical utility of that approach would require further study.

The IRM was developed and tested in European participants, due to underrepresentation of other ancestries in UKBB, which may limit the applicability of results to non-European patients. PRS distribution was higher in European individuals (Supplementary Fig. 7), likely because the variants included in the PRS were discovered through a genome-wide meta-analysis of a mostly European population; these variants will therefore be more common in Europeans. Addressing this limitation will be an important focus of future work, especially considering that the IRM may be useful for CRC risk prediction in cases of FIT noncompliance, and a recent study showed FIT uptake is lower in the following ethnic groups: Asian, Black, and mixed or other [20]. This highlights the urgent need for openly available genome-wide association study data from ancestrally diverse populations, to develop accurate PRS for more individuals, and reduce health inequalities [41, 45].

### Clinical implications

Short-term risk prediction has potential to be immediately actionable in the clinical setting. Other than the PRS, all information included in the IRM can be collected at the primary care stage. This could inform patient triage, improving early diagnosis rates and health outcomes and reducing pressure on diagnostic secondary care services.

Recent initiatives to integrate genomic data into the UK healthcare system mean that calculation of a PRS in-clinic will become feasible for increasing numbers of patients. The NHS Genomic Medicine Service, launched in October 2018, aims to routinely offer whole-genome sequencing for genetic disorders or cancer, and is considering implementation of genomic cancer screening for asymptomatic individuals in the next 5 years [46, 47]. The NHS Genomic Medicine Service is also aiming for data interoperability between NHS services, which would allow data collected for other health purposes to be used for risk stratification [47]. Other genomic healthcare programmes launched in the UK include Our Future Health, which will collect genomic and health data from 5 million adults [48], and the Newborn Genomes Programme which will sequence the genomes of >100,000 newborns [49]. These initiatives demonstrate a shift in healthcare which will increase the availability of genomic data, making implementation of a PRS for risk stratification in clinic a possibility.

A theoretical referral threshold of 0.59% risk optimises sensitivity and specificity of the IRM developed in this study. However, this is significantly lower than the >3% threshold NICE use to guide referrals [50], and the 1–2% threshold preferred by patients [42]. Diagnostic statistics for these thresholds are presented in this study, and are crucial to investigate for the optimal application of genomic data for risk prediction. Implementing a PRS or IRM in primary care should be the subject of further work.

### Conclusions

Earlier diagnosis of CRC is a priority to improve patient outcomes. Risk stratification approaches to determine which patients presenting in primary care are most likely to require diagnostic testing for CRC could increase rates of early CRC diagnosis and reduce burden on healthcare services. FIT remains the gold-standard test for prediction of CRC risk at the primary care stage. However, risk stratification methods which do not depend on FIT have the potential to improve patient outcomes in cases of patient nonadherence with the test.

The IRM developed in this study predicts, with good accuracy, which patients presenting with CRC symptoms in a primary care setting are likely to be diagnosed with CRC within the next 2 years. The IRM includes age, sex, and three symptoms (abdominal pain, rectal bleeding, and change in bowel habit). It also integrates a 201-variant PRS which stratifies patients with CRC incidence between 0.42% and 1.45% within 2 years of first symptom. The IRM was developed and tested in a mixed-sex, white European, symptomatic cohort of participants aged 40–79. Although external validation in a diverse cohort is required to test predictivity of the IRM in patients outside of this demographic, the IRM has potential to improve CRC risk prediction for the up to 16.2% of symptomatic patients noncompliant with FIT.

## Supplementary information

Supplementary Figures

Supplementary Tables

## Data Availability

This research was conducted using the UK Biobank Resource under Application Number 74981. 227 Read codes describing CRC symptoms: 200 Read codes (filtered to 151) were provided by the Diagnosis of Symptomatic Cancer Optimally (DISCO) consortium, University of Exeter. These are available upon reasonable request to the authors. Nineteen additional Read codes were found by Dr. Matthew Barclay, University College London. Using the aforementioned 170 codes as input, 57 further Read codes were identified using a function built in R (see ‘Code availability’). These codes are available from: https://github.com/bethan-mallabar-rimmer/CRC_IRM/tree/main/CRC_read_codes. 49 Read codes describing CRC: These are available from: https://github.com/bethan-mallabar-rimmer/CRC_IRM/tree/main/CRC_read_codes.

## Code availability

This analysis used an R function ‘find_read_codes’ (written by BMR, University of Exeter) which takes Read 2 and/or 3 codes as input and returns similar Read codes starting with the same series of characters. Available from: https://github.com/bethan-mallabar-rimmer/CRC_IRM/tree/main/find_read_codes. A PRS was calculated using the following R functions for analysing UKBB genetic data on the RStudio Workbench implementation on DNA Nexus, written by HG and BMR, University of Exeter: https://github.com/hdg204/Rdna-nexus. This study’s analysis pipeline is published at: https://github.com/bethan-mallabar-rimmer/CRC_IRM/tree/main.
