# Supplementary material for: Colorectal cancer risk stratification using a polygenic risk score in symptomatic primary care patients—a UK Biobank retrospective cohort study
Source: Eur J Hum Genet. 2024 Aug 1;32(11):1456–64. doi: 10.1038/s41431-024-01654-3 (PMC11577060; doi:10.1038/s41431-024-01654-3)
Supplement: Supplementary file 1 — Supplementary Figures [file 41431_2024_1654_MOESM1_ESM.docx]

**Supplementary Figure 1A – histogram and density plot of all healthcare records describing CRC in UKBB, sorted by participant age at time of record.** This includes all participant records in hospital inpatient, cancer registry, and death record data labelled with ICD-10 codes C18–20, and all GP records labelled with one of 49 Read codes for CRC (see Data Availability). Duplicated health records – from the same participant, source, and date – were de-duplicated. However, if a participant had multiple diagnoses of CRC recorded, from different sources and/or dates, these records are all included in the graph. The dashed vertical line intercepts age 40, highlighting that there are very few CRC records in UKBB from individuals younger than this at the time of the record.

**1B – distribution of age at first CRC symptom in cases and controls (no age threshold used when searching participants’ GP records for CRC symptoms).** Among participants whose first CRC symptom was reported at age <35, there are no cases with a diagnosis of CRC within two years of first symptom. The proportion of cases starts to increase among participants age 40+ at time of first CRC symptom, and increases sharply again among participants with first CRC symptom reported at age 50+.

**1C – distribution of age at first CRC symptom in cases and controls (age threshold of 40 used when searching participants’ GP records for CRC symptoms).** This age threshold improves the proportion of cases in the cohort relative to Figure 1B, as participants with a symptom at age <40 were highly unlikely to have cancer.

Abbreviations: CRC = colorectal cancer, GP = general practice, UKBB = UK Biobank.

**Supplementary Figure 2 – imputation quality metric (“info score”) across the 204 variants used to calculate PRS.** Info score ranges from 0–1. A higher info score indicates greater correlation between expected and observed genotype, and is a marker of robust imputation quality. One variant, with rsID rs2732875, was excluded from PRS calculation and this figure due to its location on the X chromosome. Three more variants (with rsIDs rs201395236, rs7038489, and rs77969132, labelled in the plot) were excluded due to having an info score <0.9. A total of 201 variants were included in the PRS.

Abbreviations: PRS = polygenic risk score.

**Supplementary Figure 3 – histogram of time difference between UKBB recruitment date (when some variables were collected at the assessment centre) and index date, across all participants in the cohort.** Variables measured at recruitment include: sex, TDI, BMI, waist circumference, smoking variables, alcohol intake, procressed meat intake, whether the participant self-reported a diabetes diagnosis, and whether the participant self-reported a parent having been diagnosed with CRC. Median time distance between recruitment date and index date was 1,603 days or 4.39 years (± interquartile range 1,956 days), shown on the plot with a dashed line.

Abbreviations: BMI = body mass index, CRC = colorectal cancer, TDI = Townsend deprivation index, UKBB = UK Biobank.

**Supplementary Figure 4 – ROCAUC of the six-variable IRM across subcohorts.** Middle bars show the ROCAUC of each model, upper and lower bars show the 95% confidence intervals. The IRM shows a trend of higher predictivity in individuals whose first CRC symptom was reported between ages 50-59 or 70-79, although there are wide confidence intervals in the 40-49 and 70-79 groups.

Abbreviations: CRC = colorectal cancer, IRM = integrated risk model, ROCAUC = receiver operating characteristic area under the curve.

**Supplementary Figure 5 – PRS distribution in cases and controls in the full cohort (top left) and subcohorts.** PRS distribution is shown using a density plot, where the area under the curve represents 100% of either the case or control population in each (sub)cohort. The dashed line shows distribution in cases, the solid line in controls. Mean PRS in each cohort is shown by vertical lines. Mean PRS was significantly higher in cases than controls (Bonferroni-corrected p-value <1.8e-03), except in all subcohorts of participants aged 40-49 and 70-79 (logistic regression p-values reported in Supplementary Table 6). Abbreviations: PRS = polygenic risk score.

**Supplementary Figure 6 – In most cohorts, ROCAUC of the six-variable IRM developed in this study is improved through inclusion of a PRS, although confidence intervals overlap.** From left to right in each cohort, the bars show ROCAUCs of 1. the PRS alone, 2. an IRM including age at first symptom, sex (only applicable in the cohorts not already stratified by sex), and symptoms of abdominal pain, change in bowel habit, and rectal bleeding, but no PRS, and 3. the combined IRM and PRS. Error bars show 95% confidence intervals. In male participants aged 60–69 and 70–79, the PRS is highly predictive – more so than the IRM without PRS. Conversely, in the non-sex-stratified cohort of participants aged 40–49, the PRS appears to decrease predictivity of the IRM. Abbreviations: IRM = integrated risk model, PRS = polygenic risk score, ROCAUC = receiver operating characteristic area under the curve.

**Supplementary Figure 7 – PRS differed across ancestries in 399,454 UKBB participants**. This is all 502,405 UKBB participants excluding 1. individuals for whom a PRS could not be calculated, for example due to lack of genotyping data (15,207 participants excluded), 2. individuals who did not cluster into any of the five ancestral populations reported here (a further 14,747 excluded), and 3. individuals who were related to the first- or second-degree to others within the same ancestral population (a further 72,997 excluded) – method reported in (37). PRS distribution in each ancestral population is shown using a density plot, where the area under the curve represents 100% of each population. A solid vertical line shows mean PRS in each population. Dashed lines show standard deviation.

A Welch’s ANOVA test (which unlike ANOVA does not assume homoscedasticity between groups) returned p <2.2e-16, showing that mean PRS differs across ancestries. Ancestral populations were compared pair-wise with Games-Howell testing (a post-hoc test for Welch’s ANOVA if group sizes are unequal, as in this cohort where non-European participants are underrepresented). This showed mean PRS in Europeans was higher than in all other groups. Mean PRS was higher in East Asian individuals than African (adjusted p = 2.90e-08) and South Asian (adjusted p = 3.58e-08) individuals; no significant difference was found compared to Admixed American participants (adjusted p = 1.21e-01).

Abbreviations: AFR = African, AMR = Admixed American, EAS = East Asian, EUR = European, PRS = polygenic risk score, SAS = South Asian.
